# Intelligence in youth and mental health at age 50

**DOI:** 10.1016/j.intell.2016.06.005

**Published:** 2016

**Authors:** Christina Wraw, Ian J. Deary, Geoff Der, Catharine R. Gale

**Affiliations:** a Center for Cognitive Ageing and Cognitive Epidemiology, Department of Psychology, University of Edinburgh, 7 George Square, Edinburgh, Scotland EH8 9JZ, United Kingdom; b MRC/CSO Social & Public Health Sciences Unit, 200 Renfield Street, University of Glasgow, Glasgow G2 3QB, United Kingdom; c MRC Life Course Epidemiology Unit, University of Southampton, Southampton General Hospital, Southampton SO16 6YD, United Kingdom

**Keywords:** Cognitive epidemiology, Intelligence, Sleep difficulties, Mental health, Depression, SES, Age 50, AFQT, NLSY-79

## Abstract

**Background:**

Few cognitive epidemiology studies on mental health have focused on the links between pre-morbid intelligence and self-reports of common mental disorders, such as depression, sleep difficulties, and mental health status. The current study examines these associations in 50-year-old adults.

**Methods:**

The study uses data from the 5793 participants in the National Longitudinal Survey of Youth 1979 cohort (NLSY-79) who responded to questions on mental health at age 50 and had IQ measured with the Armed Forces Qualification Test (AFQT) when they were aged between 15 and 23 years in 1980. Mental health outcomes were: life-time diagnosis of depression; the mental component score of the 12-item short-form Health Survey (SF-12); the 7-item Center for Epidemiological Studies Depression Scale (CES-D); and a summary measure of sleep difficulty.

**Results & conclusion:**

Higher intelligence in youth is associated with a reduced risk of self-reported mental health problems at age 50, with age-at-first-interview and sex adjusted Bs as follows: CES-depression (B = − 0.16, C.I. − 0.19 to − 0.12, *p* < 0.001), sleep difficulties (B = − 0.11, C.I. − 0.13 to − 0.08, *p* < 0.001), and SF-12 mental health status (OR = 0.78, C.I. 0.72 to 0.85, *p* < 0.001; *r* = − 0.03 *p* = 0.075). Conversely, intelligence in youth is linked with an increased risk of receiving a diagnosis of depression by the age of 50 (OR 1.11, C.I. 1.01 to 1.22, *p* = 0.024; *r* = 0.03, *p* = 0.109). No sex differences were observed in the associations. Adjusting for adult SES accounted for most of the association between IQ and the mental health outcomes, except for having reported a diagnosis of depression, in which case adjusting for adult SES led to an increase in the size of the positive association (OR = 1.32, C.I. 1.16 to 1.51, *p* < 0.001).

## Highlights

• Links between IQ in youth and four mental health outcomes at age 50 were examined.
• Higher IQ was associated with a greater risk of a depression diagnosis (OR 1.11).
• Adjusting for adult SES amplified the diagnosis of depression coefficient (OR 1.32)
• Higher IQ was associated with a lower risk of the other three mental health outcomes.

## 1 Introduction

Approximately 25% of American adults are affected by mental health problems, with anxiety disorders and depression being the most common problems (American Psychiatric Association, 2015). In 2012, both anxiety and depression were in the top 5 leading causes of disability globally (Mathers et al., 2008, Whiteford et al., 2013). In view of this, it is of public health interest to discover the factors that increase the risk of developing these disorders across the life course.

There is evidence in the literature of a link between intelligence test scores in youth and the risk of mental health difficulties in adulthood. For example, a lower IQ in childhood is associated with increased risk of schizophrenia (David et al., 1997, Osler et al., 2007, Gunnell et al., 2002, Zammit et al., 2004, Dickson et al., 2012), post-traumatic stress disorder (PTSD) (Koenen et al., 2007, Kremen et al., 2007, Gale et al., 2008, Gale et al., 2010) and depression (Zammit et al., 2004, Gale et al., 2008). Previous research has found that a SD increase of intelligence in youth tends to be associated with a 13% to 43% reduced risk of the above-mentioned mental health difficulties in adulthood (Gale et al., 2008, Gale et al., 2010, Koenen et al., 2007, Osler et al., 2007). When these studies adjusted for indicators of socio-economic status (i.e. parental occupation or education, or own education or income) the effect sizes in many cases were somewhat reduced; however, the majority of effects remained significant.

Much of this previous work was based on records of hospital admissions for mental illnesses. However, around 40% of adults in the U.S with a mental illness may not receive treatment (American Psychiatric Association, 2015). Therefore, it is important to test if this relationship between intelligence and mental disorders holds for the common, less severe cases of mental illness.

Some work in the cognitive epidemiology of mental health has focused on sub-clinical mental health problems based on self-reported measures. In two British cohorts, the 1958 National Child Development Survey and the 1970 British Cohort Study, IQ in childhood was inversely associated with risk of psychological distress in individuals in their early 30's. In these two cohorts, Childhood IQ and scores on a the Malaise depression scale correlated − 0.11 and − 0.18, respectively (Gale, Hatch, Batty & Deary, 2008). The current study aims to expand these findings by testing for links between intelligence in youth and self-reports of mental health in an American cohort of adults around the age of 50.

It is important to focus on middle-aged adults, because much of the previous work has looked at mental health problems in younger adults under the age of 40. Moreover, it is not uncommon for someone aged 45–54 to have a mental health problem (Kessler et al., 2009); for example, approximately 14% of British men and 23% of women in this age group had a common mental disorder in 2007, with similar rates for those aged 16–44 (McManus, Meltzer, Brugha, Bebbington, & Jenkins, 2009).

There is some evidence that the relationship between intelligence in youth and mental health outcomes in adulthood might be different for men and women (Hatch et al., 2007). As the current study is based on a cohort that is made up of both men and women, a sex-by-IQ interaction will be included in the analysis to test for differences in the association between intelligence and mental health outcomes in men and women.

The present study follows up on previous analyses of the NLSY-79 cohort at the age of 40 years (Der, Batty, & Deary, 2009). In that study, higher IQ in youth was significantly linked with self-reports of better overall mental health and lower levels of depression at age 40. There were also fewer self-reports of sleeping difficulties and lifetime cases of depression by the age of 40 in people with higher IQ at entry to the study. A standard deviation higher score in IQ was associated with a 23% to 12% reduced risk of having sleeping difficulties and depression, respectively, at age 40; it was also associated with approximately a fifth of a standard deviation lower depression score as well as a marginally better global mental health status score.

There are a number of ways in which the study by Der, Batty and Deary (2009) could be improved. For example, it would be useful to test how intelligence relates to both mental health status at age 50 as well as the lifetime prevalence of depression diagnosis up to age 50. In addition to this, both childhood (parental) and adult SES are associated with mental health inequalities (Wilkinson & Marmot, 2003) and intelligence (Lubinski, 2009, McLoyd, 1998) but only childhood SES was adjusted for in the models reported by Der, Batty and Deary (2009).

It is important to adjust for adulthood SES, in addition to childhood SES, as this could help to highlight a possible mediation pathway along which pre-morbid intelligence affects mental health outcomes. With regard to lower socio-economic status, it is important to mention that poverty and mental illness very likely interact dynamically, in a cycle: i.e., poverty could increase the risk of a mental illness, as explained by the social causation theory (Lund, 2012, Saraceno et al., 2005, Stansfeld et al., 2011). Mental illness might, in turn, increase the risk of poverty, as explained by the social drift theory (Dembling et al., 2002, Rodgers and Mann, 1993, Lund, 2012, Saraceno et al., 2005, Stansfeld et al., 2011, Kessler, 2004). Whereas SES is often represented by a compound index, as it is in the present study, it is also potentially informative to examine individually the impact of its components – namely, education, income, and occupational status – as possible mediators of the relationship between childhood intelligence and later health. We also note the value of having a measure of intelligence that was taken in youth, because this increases the likelihood that the measure is truly pre-morbid; it helps to avoid the possibility that poor mental health in youth confounds the association between lower IQ and poor mental health later in adulthood.

The present study also tests for associations between intelligence in youth and sleep difficulties at age 50. Although sleep difficulties are not often included in studies on mental health, they were included in the present study primarily because sleep difficulties often co-occur with psychiatric disorders such as anxiety or depression (Bixler et al., 2002, Szelenberger and Soldatos, 2005, Ford and Kamerow, 1989, Kales et al., 1983). Der et al. (2009) found that a SD increment in intelligence in youth was associated with a 23% reduction in the likelihood of reporting sleep difficulties at age 40. The current study will test this association at the age of 50 years.

The present study is particularly interested in testing how intelligence in youth is associated with different self-reported mental health outcomes (mental health status, sleep difficulties, and levels of depression) at age 50, and with self-reports of a lifetime diagnosis of depression by the age of 50. The current study also tests the role played by childhood and adult SES, and the latter's three components (education, income, and occupation status), in the above-mentioned relationships. It is hypothesized that higher IQ in youth will be associated with better mental health across all outcomes. It is hypothesized that adult SES will have a greater attenuating effect on the relationship between intelligence and mental health outcomes than childhood SES because adult SES is thought to mediate some of the association between intelligence in youth and mental health outcomes in adulthood. Finally, the present study will also test for sex-based differences in intelligence-mental health associations.

## 2 Methods

### 2.1 Participants

This study was based on data from the National Longitudinal Survey of Youth 1979 (NLSY-79). The initial sample was representative of non-institutionalized young people who lived in the United States. It was a random household sample and consisted of 12,686 individuals aged 14–21 years on 31st of December 1978. There were 6283 males (50%) and 6403 females (50%); 16% were Hispanic/Latino, 25% were Black, and 59% were non-Black & non-Hispanic; ethnic minorities were intentionally over-represented in order to obtain a large-enough sample size of these groups.

The NLSY-79 survey collected information on a variety of topics such as health, education, achievement tests, employment, and attitudes. The initial interview for NLSY-79 took place in 1979 and respondents were re-interviewed annually until 1994 and biennially thereafter. The most recent data available derive from the 2012 wave. It had a 73.3% retention rate from the initial sample to the 2012 wave, which consisted of 7301 individuals (48% males). The respondents were between 47 and 56 years of age (Bureau of Labor Statistics, 2015).

The 50 + health module was used in the present study. The data in this module were collected over three waves in 2008, 2010, and 2012. Respondents completed this module when they were approximately 50 years old (range 49–55). In total, 6893 (46%) of the initial NLSY-79 sample completed the 50 + health module (48% males). The three following measures appeared in both the 40 + health module and the 50 + health module: the 7-item Center for Epidemiological Studies Depression Scale (CES-D), the 12-item Short-Form Health Survey—mental health status (SF-12), and a question about sleep difficulty. The other question included in this study, which asked about a lifetime diagnosis of depression, was novel to the 50 + health module (NLSY-79).

### 2.2 Measures

The data were downloaded from the National Longitudinal Study (NLS) Web Investigator site on 15/11/2014 (NLSY-79).

#### 2.2.1 Intelligence

The measure of intelligence used in the NLSY-79 was the Armed Forces Qualification Test (AFQT), 1989 re-normed version. The participants in the NLSY-79 completed the AFQT in 1980 when they were aged between 15 and 23 years. This score is derived from four of the 10 subtests in the Armed Services Vocational Aptitude Battery (ASVAB). The subtests assessed the following: arithmetic reasoning (AR), mathematics knowledge (MK), word knowledge (WK), and paragraph comprehension (PC). The ASVAB has been found to be a reliable and valid measure of intelligence (Welsh, Kucinkas, & Curran, 1990). It has been found to be a predictor of academic and job performance as well as a predictor of first-term attrition rates from a military job and self-paced school completion time (Palmer et al., 1988, Welsh et al., 1990). To be consistent with the study done on the 40 + health module (Der et al., 2009), the AFQT variable used in the present study was downloaded from The Bell Curve Page (Herrnstein & Murray, 1994). This variable was scored as a percentile, and was then z-scored.

#### 2.2.2 Health outcomes

The current study examines four mental health outcomes from the 50 + health module, as follows.

##### 2.2.2.1 Lifetime diagnosis of depression

The first outcome was a self-report of ever having had a diagnosis of depression. This was elicited by the question, “Has a doctor ever told you that you have depression” and it could receive a Yes/No response.

The three further mental health outcomes were summary measures for current mental health status, levels of depression, and sleep difficulties, as follows.

##### 2.2.2.2 Mental health status

The measure of mental health status was taken from the 12-item short-form Health Survey (SF-12) (Ware, Kosinski, & Keller, 1996), which has strong validity and reliability (Resnick & Parker, 2001). This is a summary measure of the 6 questions in the SF-12 that evaluate the overall levels of mental health status at the time of testing. Scores can have a minimum value of 11.33 and a maximum value of 70.33; a higher score on this variable indicates better mental health (Ware et al., 1996). This variable was included as a dichotomous outcome because, as a continuous measure, this variable had a non-normal distribution that could not be effectively transformed to a normal distribution. This item was dichotomized at 47.74, which was the twentieth percentile of values. A score that fell below 47.74 was categorized as poor mental health and was given a value of 1. A score above this point was categorized as good mental health and received a value of 0.

In a sensitivity analysis the SF-12 was used as a continuous measure. For this, it was reversed scored to have a minimum score of 1 and a maximum score of 60, with a higher score indicating poorer mental health status. This variable was reversed scored so that its interpretation was consistent with the other outcomes and so that a higher score indicated poorer mental health status. This variable also received a square root transformation to help reduce the affect of the non-normal distribution, and was z-transformed to zero mean and unit SD.

##### 2.2.2.3 Levels of depression

The summary measure for depression was the 7-item Center for Epidemiological Studies Depression Scale (CES-D) (Radloff, 1977, Levine, 2013, Ross and Mirowsky, 1989). Each item is scored 0 to 3, and the total summed scores, therefore, potentially range from 0 to 21, with a higher score indicating more depression. The CES-D scale has strong reliability and validity (Levine, 2013, Radloff, 1977).

##### 2.2.2.4 Sleep difficulties

The sleep difficulty summary measure was made up of four questions that were taken from the Sleep Habits Question that was used in the Sleep Heart Health Study (SHHS) (Redline et al., 1998). This scale was adapted from a scale used in the Tuscan Epidemiologic Study of Obstructive Airway Disease (Klink and Quan, 1987, Quan et al., 1997, Bixler and Kales, 1979). Each question asked about frequency of different sleep problems. For example, one question asked the following, “How often do you…have trouble falling asleep?”. Each item is given a score between 1 and 4, with a higher score indicating less sleep difficulty. Responses took the form “Almost always (4 + times per week)” = score 1; “Often (2–3 times per week)” = score 2; etc. This variable was reversed scored and the scale went from 1 to 13; this was done so that a higher score indicated more sleep problems.

#### 2.2.3 Covariates

Potential confounding and mediating variables were as follows: age at initial interview when recruited to NLSY-79, adult age when the health at 50 module was completed, sex, childhood SES, and adult SES (along with its three constituent parts of education, income, and occupational status). We now describe how these variables were created.

Childhood SES was a z-transformed composite variable of parental income, education, and occupation status, which was derived by Herrnstein and Murray (1994). Higher scores on the childhood SES variable indicate a more advantaged socio-economic position (Herrnstein & Murray, 1994). The adult SES variable was also a derived variable. The method used to make this variable was similar to that used by Herrnstein and Murray (1994) to derive childhood-SES. In other words, adult SES is an average of z-scored adult educational attainment, income, and occupation status.

For adult educational attainment the variable ‘Highest Grade Completed’, as of 2012 was used and was z-transformed. The variable for income was ‘Total Net Family Income In Past Year’. Income received a square root transformation prior to a z-transformation. The third component of the adult SES variable was occupation status. This was coded according to the US 3-digit, 2000 census code; this is explained in NLSY-79 Attachment 1: Census Industrial & Occupational Classification (Bureau of Labor Statistics, 2015). This was then used to derive an Occupational status hierarchy. Herrnstein and Murray (1994) used the 1960 Duncan socio-economic index (SEI) scale but, because many changes had been made to the census occupation classification system between 1980 and 1990 (Frederick, 2010), an updated version of the 1960 Duncan SEI scale was used here. This scale was developed by Hauser and Warren (1996) and was constructed in a similar way to the Duncan SEI (Frederick, 2010). Occupation status received a log transformation and was then z-scored. Higher scores on the composite adult SES variable and on each of its constituent components indicate more advantaged socio-economic position.

### 2.3 Analysis

Two sets of analyses were conducted. The primary set of analyses was performed using a complete case analysis. The other set of analyses was a multiple imputations analysis. Each set of analyses comprised a series of hierarchical regression analyses. Logistic regressions were conducted for diagnosed depression and the dichotomized SF-12 mental health status score. Generalized linear models were applied for sleep difficulty and CES-Depression, because these outcomes were both heavily skewed to the right. Both models assumed a gamma distribution and used a log link.

For each mental health outcome, six separate models were analyzed. The baseline model adjusted for age at NLSY-79 baseline (when AFQT was tested) and sex. Model 2 additionally adjusted for childhood SES. Model 3 added composite adult SES to the variables included in Model 2. Models 4, 5, and 6 were the same as Model 3 but each replaced the composite adult SES with one of its constituents: income, education, or occupational status. A sex by IQ interaction was tested in all models. All analyses were conducted in STATA 13.0.

#### 2.3.1 Sensitivity analyses

A second set of analyses was performed using multiple imputations to the data. The imputed analysis was selected due to the relatively high proportion of missing data for the adult SES variable. Twenty-eight imputations were created using the multivariate normal regression method for arbitrary patterns of missing data. The variables imputed were income, education, and occupation status, because these had the highest rates of missing data, with 21%, 6% and 14% missing, respectively. Twenty-eight imputations were selected, because 28% of the composite-Adult SES values were missing. Imputations were conducted on only those who were present for the 50 + health module.

As an additional check of the effect of intelligence in youth on sleep difficulty and CES-Depression, logistic sensitivity models were run on these outcomes. To run a logistic model, both scales had to be dichotomized. For CES-Depression, the accepted cut-off point for depression of > 8 was used to create a dichotomous variable (Levine, 2013). As the sleep difficulty scale used in the present study was a subset of a larger scale and no standard cutoff point was known, the top of the first quartile was used as a cut-off point, and a score < 11 was used to indicate that a sleep problem was present. A linear regression was also conducted with the continuous SF-12 mental (health) measure as part of the sensitivity analysis.

An additional set of complete case analyses was conducted which was weighted for ethnicity to correct for the over representation of ethnic minorities in the NLSY-79.

The non-weighted complete case analyses are the primary focus of the results section. The results from the imputed analysis are briefly covered at the end of the results, and are covered in more detail as Supplementary material.

## 3 Results

### 3.1 Descriptive statistics

The present analysis was based upon a sample of 5793 participants who responded to the 50 + health module. There were 1619 missing values for adult SES, 1239 were missing for income, 818 for occupational status, and 339 for education. For IQ, 293 were missing. The complete cases analysis was conducted on those 4015 respondents who had complete data for IQ, age, sex, educational attainment, occupational status, and adult income. The numbers used for each analysis vary slightly from this due to small numbers (< 3%) of missing data for the health outcomes. Table 1 shows differences in selected characteristics between those who did and did not complete the 50 + health module. Those who completed it were significantly more likely to be female, had slightly lower IQ scores (− 0.04 of a SD) and were significantly older (by a mean of 1.29 years) than those who did not complete the 50 + health module. There were no significant differences between the two groups in income, education, occupation status, or adult SES.

Among the 4015 participants who completed the 50 + health module included in the complete case analysis, there was a significant and positive association between IQ and childhood SES (*r* =.56, *p* <.001), adult SES (*r* =.64, *p* <.001), and the sub-components of Adult SES, which includes income (*r* =.49, *p* <.001), education (*r* =.60, *p* <.001), and occupational status (*r* =.48, *p* <.001) (Table A.1 Appendix A). Pairwise correlations between AFQT and CES-depression and SF-12 mental health status were run to estimate the strength of the true relationship, with adjustment for the reliability of these measures. The parallel forms reliability coefficients of AFQT was 0.92 (Welsh et al., 1990), and the internal consistency alpha coefficient of SF-12 mental health status and CES-depression was 0.80 (Resnick & Parker, 2001) and 0.82 (Levine, 2013), respectively. The correlations between AFQT and SF-12 mental health status increased from −.028 to −.033 and the correlation between AFQT and CES-depression increased from −.139 to −.160 (Table A.5).

Table 2 displays the descriptive statistics for the four mental health outcome.

variables. The mean for CES-depression, SF-12 mental (health) status, and sleep difficulties was significantly higher for women than for men. The standard deviation was greater in all three outcomes for women than it was for men. A greater percentage of women (22%) had a lifetime diagnosis of depression than men (10%).

Table 3 displays the result of the generalized linear regression analysis of CES-depression and sleep difficulty regressed on IQ. When age at first interview and sex were adjusted for in the initial model, higher IQ was significantly associated with lower scores for depression and sleep difficulties. For CES-Depression a one standard deviation higher score in IQ was associated with B^1^ = − 0.16 (95% C.I. − 0.19 to − 0.12, *p* <.001). For sleep difficulty a one standard deviation higher score in IQ was associated with B = − 0.11 (95% C.I. − 0.13 to − 0.08), *p* <.001. When childhood SES was included there was only slight attenuation in the effect size for the relationship between IQ and sleep difficulty and CES-Depression, and both associations remained significant. Including adult age and adult SES in Model 3 had a substantial attenuating effect on associations between IQ and CES-Depression and Sleep Difficulty, which became non-significant. The sex by IQ interaction was not significant for any of these continuous mental health outcomes. For example, the sex by IQ interaction in the initial model for sleep difficulty and CES-depression had B = 0.01 (*p* =.68), and B = 0.03 (*p* =.30), respectively.

Table 3 also displays the result of the logistic regression for the relationship between IQ and self-reported diagnosis of depression and the dichotomized SF-12 mental health status variable. In the baseline model, IQ was significantly and negatively associated with SF-12 mental health status and was nominally significantly and positively associated with a doctor diagnosis of depression. A one standard deviation higher score in IQ was associated with reduced odds of having poor mental health status, with an odds ratio of 0.78 (95% C.I. 0.72 to 0.85, *r* = −.03). A one standard deviation higher score in IQ was associated with increased odds of lifetime diagnosis of depression, with an odds ratio of 1.11 (95% C.I. 1.01 to 1.22, *r* =.03). Including childhood SES led to the attenuation of the effect size for a doctor diagnosis of depression to non-significant levels, but it did not attenuate the effect size for SF-12. When adult SES was included, the association between IQ and SF-12 mental health status was almost wholly attenuated and was no longer significant. Including adult SES led to an increase in effect size for doctor diagnosis of depression and resulted in an odds ratio of 1.32 (95% C.I. 1.16 to 1.51). The sex by IQ interaction was not significant for either of these dichotomized mental health outcomes. The sex by IQ interaction in the initial model for SF-12 and diagnosis of depression was OR 1.07 (*p* =.36) and OR 1.10 (*p* =.29), respectively.

**Fig. 1:**
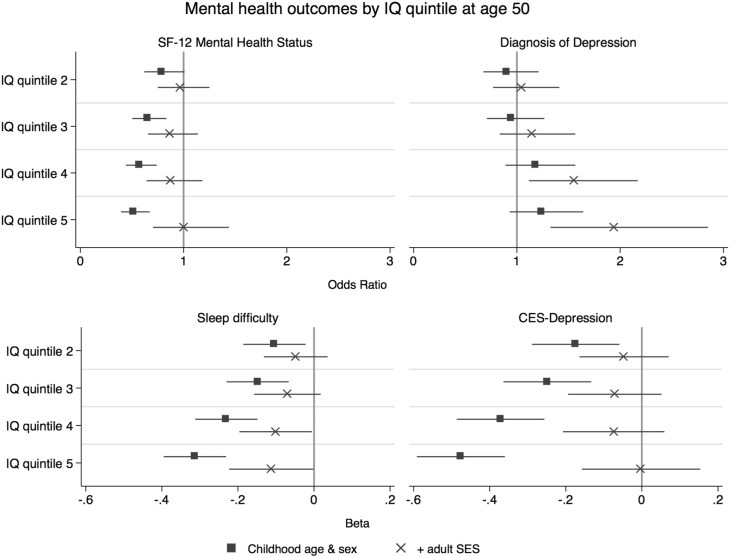
Odds ratio and beta regression coefficients (with 95% confidence intervals) for four mental health outcomes per AFQT quintile at age 50, with adjustment for childhood age and sex and then further adjustment for adult SES. Note SF-12 mental health status = 12-item short-form health survey (mental health component).

The results of the regression analysis that adjusted for the three sub-components of adult SES (income, education, and occupation status) for all of the mental health outcomes in the complete case analyses are presented in Table A.2 in the Appendix. This table shows how IQ in youth is associated with the four different mental health outcomes when income, education, and occupation status are adjusted for separately. Adjusting for the composite adult SES variable led to greater attenuation of the associations between IQ and CES-depression, sleep difficulty, and SF-12 mental health status than adjusting for income, education, and occupation status individually. Of the three adult SES components, adjusting for income led to more attenuation of the associations between IQ and CES-depression, sleep difficulty, and SF-12 mental health status than did education and occupation status. Looking at the self-report diagnosis of depression, adjusting for the composite adult SES led to more amplification of the positive association with IQ than income, education, or occupation status did individually. Much of the amplification effect of adjusting for adult SES was also found when adjusting for income. Fig. 1 displays the results of the four mental health outcomes by IQ quintile for the two models that adjust for age at first interview and sex, and additionally for adult SES, in the complete case analyses. Table 4 shows descriptive statistics of AFQT score broken into quintiles.

The complete case analyses were repeated, weighted for ethnicity (Table A.6) and overall the results were similar to the original complete case analysis. The most notable deviation in results was that the association between intelligence in youth and a lifetime diagnosis of depression was not significant in the weighted analysis, in the baseline model. However, in the other five models that analyzed the association between intelligence in youth and a lifetime diagnosis of depression, the effect sizes and significance levels in the weighted analysis were similar to those in the complete case analyses. One other notably different result was that the strength of the association between intelligence and sleep difficulties increased and became significant after adjusting for adult SES. Weighting for race and ethnicity, at most, only marginally influenced all of the other results across the four mental health outcomes.

The results for the complete cases analysis were also compared to the results from the multiply imputed analysis (Table A.3 on the appendix). The overall patterns were similar between the imputed and complete case analysis across the four mental health outcomes. A detailed description of these results can be found in the supplementary materials. The results of the logistic and linear sensitivity analyses of the relationship between intelligence in youth and CSE-depression, sleep difficulty, and SF-12 mental health status were similar to those found in the complete case analysis. These results are described in the supplementary materials.

## 4 Discussion

The present study examined the relationship between IQ in youth and four mental health outcomes taken at age 50. It also examined how associations changed after adjusting for childhood and adult SES. The results suggest that pre-morbid intelligence was significantly associated with mid-life mental health outcomes. In the baseline model that adjusted for age at IQ test and sex, a higher pre-morbid IQ was associated with less CES-depression, less sleep difficulty, and better SF-12 mental health status at age 50. In contrast, a higher pre-morbid intelligence was associated with higher rates of a lifetime diagnosis of depression. Adjusting for childhood SES had little attenuating effect. Adjusting for adult SES led to substantial attenuation of IQ's association with CES-depression, sleep difficulty, and SF-12 mental health, but it amplified the effect size of the association with a diagnosis of depression. No significant sex differences were found in the relationship between IQ in youth and any of the mental health outcomes, which is consistent with other studies (Gale et al., 2009, Mikkelsen et al., 2014, Johnson et al., 2011).

The present study provides a novel contribution to our understanding of the association between intelligence in youth and adult mental-health. It is one of the few studies to report associations between intelligence and both a history of depression diagnosis and current self-reported mental health problems in both men and women in mid-life, around age 50 years. Higher pre-morbid intelligence was significantly associated with less depression, less sleep difficulty, and a better overall mental health status at age 50. These results were similar to those found at age 40 (Der et al., 2009) and they suggest that higher intelligence in youth, in both men and women, may have a protective effect on mental health into middle age.

An intriguing aspect of the results is the contrast in the results between CES-depression scores and a lifetime diagnosis of depression. The results suggest that higher intelligence is linked with lower rates of depression at age 50 but with a higher lifetime diagnosis of depression. This suggests that those with higher intelligence in youth are at an increased risk of being diagnosed with depression by the age of 50. Two other studies reported a positive relationship between cognitive ability and lifetime cases of depression (Luciano et al., 2015, Cullen et al., 2015). A small number of studies have also found some evidence that suggests there may be a positive correlation between IQ and increased risk of bipolar disorder (Gale et al., 2013, Higier et al., 2014, Smith et al., 2015) and mania in adulthood (Koenen et al., 2009). However, the current findings on depression diagnosis do deviate from the general trend in the literature which tends to find higher intelligence is associated with less mental illness (Der et al., 2009, Gale et al., 2008, Martin et al., 2007, Weeks et al., 2014).

One possible explanation for the positive association between intelligence and lifetime diagnosis of depression in the current study could be health literacy. Evidence suggests health literacy is strongly associated with general intelligence (Reeve and Basalik, 2014, Gottfredson, 2004, Mottus et al., 2014, Murray et al., 2011, Wolf et al., 2012). Following from this, people with higher intelligence may also have higher mental health literacy. Those with higher intelligence might be more able to identify their symptoms of depression, which could motivate them to consult a doctor for diagnosis and advice; they might also be likely to have accurate reporting of such diagnoses in the health module (Beier and Ackerman, 2003, Gottfredson, 2004). Conversely, people with a lower IQ might not be aware of symptoms of depression and may therefore be less likely to become diagnosed with depression and therefore less likely to report a diagnosis of depression than people with higher levels of intelligence.

Another possible explanation for the positive association between IQ and a reported diagnosis of depression, as observed in the current study, could relate to incentives for more intelligent people to seek out a diagnosis of depression in the United States. It is possible that more intelligent Americans are motivated to strategically get a diagnosis of depression if they experience symptoms of psychological distress, even if they are not actually depressed, because it would allow them to get insurance coverage for treatment, which they might think they would benefit from (APAPO, 2002). As intelligence is strongly correlated with social class, evidence for this may be found by referring to socio-economic class differences in mental health treatment. In the US the higher social classes are disproportionally represented among those receiving privately-funded mental health treatment (Hollingshead & Redlich, 2007). Higher social class individuals are also more likely to receive psychotherapy as their treatment, whereas lower class individuals are more likely to receive biological treatment (i.e. drugs or electro-shock therapy) for the same condition (Hollingshead & Redlich, 2007). This might highlight a deterrent for people from a lower social class to seek out a diagnosis of depression, as their treatment is likely to be more invasive. The less invasive psychotherapy treatment, on the other hand, might act as an incentive for people from a higher social class to seek out a diagnosis. This hypothesis could be used to guide future studies that seek to analyze the interplay between intelligence, social class, and lifetime diagnosis of depression.

Childhood SES was found to explain at most a small part of the relationship between intelligence in youth and each of the mental health outcomes, which is similar to the results found in other studies (Der et al., 2009, Koenen et al., 2007; Gale, Hatch, et al., 2008; Martin et al., 2007, Weeks et al., 2014, Wrulich et al., 2014). This suggests that the relationship between intelligence in youth and mental health outcomes is not confounded by childhood SES.

Adult SES accounted for much of the relationship between pre-morbid intelligence and CES-depression, sleep difficulty, and SF-12 mental health status at age 50. After adjusting for adult SES the betas for CES-depression and sleep difficulty were both reduced. The odds ratio for SF-12 mental health status was also reduced. None of these effects remained significant. The composite adult SES variable had a greater attenuating effect than income, education, and occupation status did on the association between IQ in youth and all three of these outcomes. Income had a greater attenuating effect than education and occupation status and therefore explained much, but not all of, the attenuation by adult SES. Other studies had similar findings. Wrulich et al. (2013) found similar results in their study. They found that the combination of adult SES and education partially mediated the effect of intelligence in youth on functional health and overall subjective health (as measured by a rating of their overall health status, their health status compared to peers, and their overall satisfaction with their health) in adulthood, reducing the standardized regression coefficients from beta = 0.18 and beta = 0.11 to beta = 0.07 and beta = 0.06, respectively. One possible explanation for this could be that stress is a well-established determinant of mental health problems. Individuals with a higher IQ are more likely to be in a higher social class and make more money, and therefore tend to experience less stress (Marmot, 2004, Sapolsky, 2005), as IQ and these indicators of SES are strongly correlated.

Adjusting for adult SES led to an increase in effect size between pre-morbid intelligence and a lifetime diagnosis of depression. The odds ratios went from 1.11 in the age and sex adjusted model to 1.32. The composite adult SES variable had a greater strengthening effect than income, education, and occupation status did. Income had a greater strengthening effect than either education or occupation status; therefore, much of the impact of adult SES is explained by income. This suggests that income in adulthood may facilitate the impact of intelligence in youth on the risk of ever having a lifetime diagnosis of depression. This might indicate that people with high intelligence, but a low income, might be less likely to receive a diagnosis of depression. This may also be explained by the US health care system and the need for health insurance, whereby higher income might afford a greater likelihood of presenting to private health care and obtaining a diagnosis of depression.

### 4.1 Strengths & limitations

The current research has some strengths. The first is that this study utilises a measure of intelligence that is taken in youth, and has an almost 30-year gap between that and the mental health measures. Youth is an optimal time for cognitive measurement, as it is towards the end of most fluid cognitive maturation and prior to any expected age-associated cognitive decline. It reduces the likelihood of reverse causation, whereby mental health might affect IQ test performance. By adolescence, shared environmental influences on intelligence have almost disappeared and much of the observed differences in intelligence are due to genetic factors (Haworth et al., 2010). A second strength of this study is that it is based on a large and representative sample of adult Americans aged 50 years old. Middle age adults are underrepresented in mental health literature, including the mental health literature that looks at intelligence as a risk factor. Much of the work in this area has focused on mental health in youth and adults under the age of 40. This is important because mental health problems are known to be an issue at middle age (McManus et al., 2009). Another strength is that unlike other studies in the field that studied IQ's associations with mental health, the present study was based on a sample that includes women. A further strength of this study is that it included a question on lifetime diagnoses of depression. Regular self-report questions tend to have poorer agreement with medical records when the condition of interest has vaguely-defined diagnostic criteria than when it has clearly defined diagnostic criteria (Haapanen, Miilunpalo, Pasanen, Oja, & Vuori, 1997). Explicitly asking whether or not respondents have ever received a clinical diagnosis of depression, instead of just relying on retrospective self-report, reduces the risk of inaccurate recall and recall bias (Coughlin, 1990, Newman and Bland, 1998).

This study also has some limitations. First, there are a high number of missing data for the adult SES variable. Because of this, the number of observations used in the complete cases analysis was reduced, though still large. The mental health measures in this study are self-reported. Such measures tend to have lower validity than clinically diagnosed conditions in medical records. However, the scales used in the study do have high reliability and validity (Resnick and Parker, 2001, Levine, 2013, Radloff, 1977, Bixler and Kales, 1979). An additional limitation is that this study does not include a measure of mental health that was taken at the same time as the measure of intelligence. This is not available in the current data set, but would have been useful to have as a covariate because it could have adjusted for the potential effect of poorer mental health earlier in life possibly influencing IQ test performance, and also leading to lower social status in adulthood. The current study also did not account for potential intervening variables that might mediate the relationship between intelligence in youth and mental health outcomes at age 50. Future research in this area may benefit from looking at how possible intervening variables, such as stressful life events (parental divorce, death in the family, or hospitalization etc.) or physical illness might influence the relationships observed in the present study. There is also the possibility of reporting bias due to differences in mental health literacy (Beier and Ackerman, 2003, Gottfredson, 2004), whereby those with higher intelligence might have better symptom recognition, better recall of conditions, and more accurate reporting of these in the health module. The net effect of such reporting bias would be to bias some results towards the null, i.e. our estimates of effect size here might be under-estimates.

The findings of this study demonstrate that there is a relationship between intelligence in youth and mental health in middle age. The results suggest that higher intelligence in youth is linked with lower rates of self-reported mental health problems at age 50. Adjusting for adult SES seems to substantially attenuate the association between IQ and depression and sleep problems at age 50. The current study also found that higher intelligence is linked with higher rates of receiving a diagnosis of depression by 50 years of age. These findings may be due to an artifact and may be explained by those with lower intelligence not receiving a diagnosis of depression when they are depressed and/or not seeking a diagnosis due to fear of stigma. If this is the case, then this study provides evidence that there is a need to tailor mental health interventions for those with lower intelligence to help increase symptom recognition of depression and to help them overcome stigma surrounding depression. The current findings contribute to our understanding of how intelligence is related to mental health. Higher intelligence seems to help reduce the risk of mental health problems. These findings can be used to help inform mental health policy as well as mental health interventions that target mental illness in different sub-groups of the population.

## Figures and Tables

**Table A.1:** Pairwise correlation matrix for outcome variables, explanatory variables, and covariates for men and women.

	IQ	Adult SES	Income	Education	Occupation status	Childhood SES	CES-depression	SF-12 mental health status	Sleep difficulty	Diagnosis of depression
IQ	1									
Adult SES	.643	1								
<.001									
Income	.488	.783	1							
<.001	<.001								
Education	.603	.828	.455	1						
<.001	<.001	<.001							
Occupation Status	.479	.830	.461	.564	1					
<.001	<.001	<.001	<.001						
Childhood SES	.562	.467	.363	.450	.327	1				
<.001	<.001	<.001	<.001	<.001					
CES-Depression	−.139	−.221	−.261	−.135	−.143	−.080	1			
<.001	<.001	<.001	<.001	<.001	<.001				
SF-12 Mental Health Status	−.028	−.109	−.162	−.050	−.056	.005	.658	1		
.075	<.001	<.001	.002	<.001	.769	<.001			
Sleep Difficulty	−.131	−.164	−.183	−.124	−.094	−.076	.537	.415	1	
<.001	<.001	<.001	<.001	<.001	<.001	<.001	<.001		
Diagnosis of Depression	.025	−.057	−.120	−.006	−.012	.035	.397	.418	.312	1
.109	<.001	<.001	.685	.437	.026	<.001	<.001	<.001	

**Table A.2:** Linear and logistic* regression analyses of the relation between a SD increase in IQ in youth and five mental health outcomes by age 50, with adjustment for potential confounding or mediating variables, across Model 4–Model 6 of the complete case analysis.

	Model 4			Model 5			Model 6		
+ Income			+ Education			+ Occupation status		
	95% C.I.	*P* value		95% C.I.	*P* value		95% C.I.	*P* value
	Beta			Beta			Beta		
CES-depression	− 0.03	− 0.08 to 0.02	.181	− 0.09	− 0.14 to − 0.04	.001	− 0.11	− 0.15 to − 0.06	<.001
Sleep difficulty	− 0.05	− 0.08 to − 0.02	.003	− 0.06	− 0.10 to − 0.03	.001	− 0.09	− 0.12 to − 0.05	<.001
	OR			OR			OR		
SF-12 mental health (dichotomous)	0.96	0.86 to 1.08	.509	0.84	0.75 to 0.95	.004	0.83	0.74 to 0.92	.001
Diagnosis of depression	1.31	1.16 to 1.48	<.001	1.16	1.02 to 1.31	.023	1.11	0.99 to 1.25	.087

Note.

The effect of IQ on mental health, CES-depression, and sleep difficulty was analyzed using linear regression analysis, Beta = regression coefficient.

The effect of IQ on depression and emotional/nervous disorders was analyzed using logistic regression, Beta = odds ratio.

Sample size: mental health (3985), depression (3981), sleep difficulty (3992).

Observations (Yes): depression 4004(564), emotional/nervous disorder 4010 (330).

Model 4: IQ, Sex, Childhood age, Adult age, Childhood SES, Income.

Model 5: IQ, Sex, Childhood age, Adult age, Childhood SES, Education.

Model 6: IQ, Sex, Childhood age, Adult age, Childhood SES, Occupation status.

**Table A.3:** Linear and logistic regression analyses of the relation between a SD higher score in IQ in youth and five mental health outcomes at age 50, with adjustment for potential confounding or mediating variables across Model 1–Model 6 (imputed analysis).

	Model 1			Model 2			Model 3		
	95% C.I.	*P* value		95% C.I.	*P* value		95% C.I.	*P* value
	Beta			Beta			Beta		
SF-12 mental health (continuous)^a^	−.07	−.10 to −.05	<.001	−.09	−.12 to −.06	<.001	−.02	−.05 to .02	.386
CES-depression	− 0.19	− 0.22 to − 0.16	<.001	− 0.19	− 0.22 to − 0.15	<.001	− 0.07	− 0.11 to − 0.03	.001
Sleep difficulty	− 0.12	− 0.14 to − 0.10	<.001	− 0.11	− 0.14 to − 0.09	<.001	− 0.05	− 0.08 to − 0.02	.001
	OR			OR			OR		
SF-12 mental health (dichotomous)	0.71	0.67 to 0.76	<.001	0.71	0.66 to 0.78	<.001	0.85	0.78 to 0.94	.001
Diagnosis of depression	1.01	0.94 to 1.09	.771	0.99	0.90 to 1.08	.740	1.18	1.07 to 1.31	.001

	Model 4			Model 5			Model 6		
	95% C.I.	*P* value		95% C.I.	*P* value		95% C.I.	*P* value
	Beta			Beta			Beta		
SF-12 mental health (continuous)^a^	−.03	−.07 to .002	.069	−.06	−.09 to −.02	.002	−.06	−.10 to −.03	<.001
CES-depression	− 0.10	− 0.14 to − 0.07	<.001	− 0.13	− 0.17 to − 0.09	<.001	− 0.14	− 0.18 to − 0.11	<.001
Sleep difficulty	− 0.07	− 0.10 to − 0.04	<.001	− 0.07	− 0.10 to − 0.04	<.001	− 0.10	− 0.12 to − 0.07	<.001
	OR			OR			OR		
SF-12 mental health (dichotomous)	0.83	0.76 to 0.91	<.001	0.76	0.70 to 0.84	<.001	0.76	0.70 to 0.83	<.001
Diagnosis of depression	1.15	1.04 to 1.27	.004	1.08	0.98 to 1.19	.125	1.04	0.94 to 1.14	.468

a Standardized beta.

**Table A.4:** Linear and logistic regression analysis of the relationship between a SD higher score in IQ in youth and continuous SF-12 health status and dichotomized CES-depression and sleep difficulty scale at age 50, with adjustment for potential confounding and mediating variables across Model 1–6 in the complete case analysis.

	Model 1			Model 2			Model 3		
Childhood age & sex			+ Childhood SES			+ Adult age & adult SES		
	95% C.I.	*P* value		95% C.I.	*P* value		95% C.I.	*P* value
	Beta			Beta			Beta		
SF-12 mental health (continuous)^a^	−.03	−.06 to .01	.117	−.04	−.08 to −.01	.022	.05	.01 to .09	.026
	OR			OR			OR		
CES-Depression	0.72	0.65 to 0.79	<.001	0.73	0.65 to 0.81	<.001	0.98	0.86 to 1.11	.748
Sleep Difficulty	0.73	0.67 to 0.79	<.001	0.74	0.67 to 0.81	<.001	0.86	0.77to 0.97	.011

	Model 4			Model 5			Model 6		
+ Income			+ Education			+ Occupation status		
	95% C.I.	*P* value		95% C.I.	*P* value		95% C.I.	*P* value
	Beta			Beta			Beta		
SF-12 mental health (continuous)^a^	.03	−.01 to 0.07	.099	−.01	−.05 to .04	.792	−.02	−.06 to .02	.415
	OR			OR			OR		
CES-Depression	0.93	0.82 to 1.04	.200	0.83	0.73 to 0.94	.003	0.80	0.71 to 0.90	<.001
Sleep Difficulty	0.87	0.78 to 0.97	.009	0.79	0.71 to 0.88	<.001	0.75	0.68 to 0.83	<.001

a Standardized beta.

**Table A.5:** Correlation with adjustment for reliability.

	IQ	SF-12 mental health status	CES-depression
IQ	1		
SF-12 mental health status	−.033	1	
CES-depression	−.160	.812	1

**Table A.6:** Linear and logistic regression analyses of the relation between a SD higher score in IQ in youth and five mental health outcomes at age 50, with adjustment for sampling weights as well as potential confounding or mediating variables across Model 1–Model 6.

	Model 1			Model 2			Model 3		
	C.I.	*P* value		C.I.	*P* value		C.I.	*P* value
Self-report scale at age 50	Beta			Beta			Beta		
SF-12 mental health (continuous)	− 0.02	− 0.06 to 0.01	.205	− 0.03	− 0.07 to 0.01	.145	0.05	− 0.001 to 0.09	.057
CES-depression	− 0.20	− 0.25 to − 0.15	<.001	− 0.17	− 0.22 to − 0.11	<.001	− 0.02	− 0.08 to 0.05	.646
Sleep difficulty	− 0.13	− 0.16 to − 0.10	<.001	− 0.12	− 0.16 to − 0.08	<.001	− 0.13	− 0.17 to − 0.09	<.001
	OR			OR			OR		
SF-12 mental health (dichot)	0.78	0.71 to 0.87	<.001	0.80	0.71 to 0.90	<.001	0.96	0.84 to 1.09	.509
Diagnosis of depression	1.03	0.92 to 1.15	.583	1.01	0.89 to 1.16	.858	1.27	1.09 to 1.47	.002

	Model 4			Model 5			Model 6		
	C.I.	*P* value		C.I.	*P* value		C.I.	*P* value
Self-report scale at age 50	Beta			Beta			Beta		
SF-12 mental health (continuous)	0.04	− 0.01 to 0.08	.111	− 0.01	− 0.05 to 0.04	.798	− 0.02	− 0.06 to 0.03	.484
CES-depression	− 0.05	− 0.11 to 0.002	.060	− 0.11	− 0.18 to − 0.05	.001	− 0.13	− 0.19 to − 0.07	<.001
Sleep difficulty	− 0.08	− 0.12 to − 0.04	<.001	− 0.08	− 0.13 to − 0.04	<.001	− 0.11	− 0.15 to − 0.07	<.001
	OR			OR			OR		
SF-12 mental health (dichot)	0.96	0.84 to 1.09	.482	0.85	0.74 to 0.96	.011	0.82	0.72 to 0.93	.002
Diagnosis of Depression	1.25	1.08 to 1.440	.002	1.09	0.95 to 1.26	.225	1.06	0.92 to 1.22	.405

**Table 1:** Differences in selected variables between those who completed the 50 + health module and those who did not.

	Completed 50 + health module	Obs (%)	Mean	SD	Mean difference	t	χ^2^	p*
AFQT (IQ)	Yes	5500	− 0.28	1.03	− 0.04	2.20		.03
No	6378	− 0.24	1.00	
Age (2012)	Yes	5793	51.60	1.75	1.29	− 32.66		<.001
No	6893	50.31	2.54	
Income	Yes	4705	$75,628	$82,220	−$264	0.11		0.91
No	1602	$75,892	$83,077	
Education	Yes	5454	13.20	2.60	− 0.03	0.37		0.71
No	1836	13.23	2.60	
Occupation status	Yes	4975	35.87	13.68	− 0.44	1.14		0.26
No	1708	36.31	13.91	
Adult SES	Yes	4174	0.06	0.81	− 0.01	0.45		0.65
No	1445	0.06	0.81	
Sex	Yes	5793					26.68	<.001
Male	2779 (48)		
Female	3014 (52)		
No	6893				

Note.

⁎ *p*-Value for the difference between groups.

**Table 2:** Descriptive statistics for the mental health outcome variables.

	Obs (%)	Mean	SD	Min	Max	Mean difference	t	χ^2^	*p*
AFQT (IQ)									
Male	2608	− 0.23	1.08	− 3	3	0.083	2.98		.003
Female	2892	− 0.32	0.98	− 3	3
Age									
Male	2779	49.76	0.77	49	55	− 0.001	− 0.04		.97
Female	3014	49.76	0.75	49	55
Adult SES									
Male	2019	0.07	0.83	− 2.21	2.86	0.03	1.14		.25
Female	2155	0.04	0.79	− 2.53	2.85	
Income									
Male	2256	$82,135	89,450	0	$497,763	$12,502	5.23		<.001
Female	2449	$69,633	74,463	0	$497,763	
Education									
Male	2585	13.04	2.55	2	20	− 0.31	− 4.45		<.001
Female	2869	13.35	2.63	3	20	
Occupation status									
Male	2397	35.40	13.60	9.56	80.5	− 0.90	− 2.32		.02
Female	2578	36.30	13.74	9.56	80.5	
CES-depression									
Male	2745	3.30	4.10	0	21	− 1.23	− 10.40		<.001
Female	2989	4.53	4.79	0	21	
SF 12: mental health status (continuous)									
Male	2752	17.46	8.26	3.87	58.41	− 2.21	− 9.33		<.001
Female	2977	19.67	9.57	1	60	
SF 12: mental health status poor									
Male								58.74	<.001
No	2317 (84)						
Yes	435 (16)						
Female							
No	2265 (76)						
Yes	712 (24)						
Sleep difficulty									
Male	2752	3.58	3.24	1	13	− 0.97	− 10.80		<.001
Female	2991	4.55	3.55	1	13	
Diagnoses of depression									
Male								152.48	<.001
Yes	274 (10)						
No	2498 (90)						
Female							
Yes	656 (22)						
No	2348 (78)						

**Table 3:** Linear and logistic regression analyses of the relation between a SD higher increase in IQ in youth and five mental health outcomes at age 50, with adjustment for potential confounding or mediating variables, across Model 1–Model 3 of the complete case analysis.

	Model 1			Model 2			Model 3		
Childhood age & sex			+ Childhood SES			+ Adult age & adult SES		
	95% C.I.	*P* value		95% C.I.	*P* value		95% C.I.	*P* value
	Beta			Beta			Beta		
CES-depression	− 0.16	− 0.19 to − 0.12	<.001	− 0.16	− 0.20 to − 0.11	<.001	0.01	− 0.05 to 0.05	.853
Sleep difficulty	− 0.11	− 0.13 to − 0.08	<.001	− 0.10	− 0.14 to − 0.07	<.001	− 0.03	− 0.07 to 0.003	.073
	OR			OR			OR		
SF-12 mental health (dichotomous)	0.78	0.72 to 0.85	<.001	0.78	0.70 to 0.86	<.001	0.98	0.87 to 1.10	.704
Diagnosis of depression	1.11	1.01 to 1.22	.024	1.05	0.94 to 1.18	.356	1.32	1.16 to 1.51	<.001

Note. The effect of IQ on SF-12 mental (health), CES-depression, and sleep difficulty was analyzed using linear regression analysis, Beta = regression coefficient.

The effect of IQ on depression and emotional/nervous disorders was analyzed using logistic regression.

Sample size: SF-12 mental (health) (3985) depression (3981), sleep difficulty (3992).

Observations (Yes): depression 4004(564), emotional/nervous disorder 4010 (330).

Model 1: IQ, Sex, Childhood age.

Model 2: IQ, Sex, Childhood age + Childhood SES.

Model 3: IQ, Sex, Childhood age, Adult age, Childhood SES + Adult SES.

**Table 4:** AFQT score by quintile.

AFQT by quintile				
Quintile	Obs	AFQT		
Mean	Min	Max
1	803	− 1.56	− 3.00	− 1.04
2	804	− 0.73	− 1.04	− 0.46
3	802	− 0.21	− 0.46	0.06
4	804	0.37	0.06	0.71
5	802	1.27	0.71	3.00

## Appendix A

Complete case analysis: Model 4, 5, and 6.

Imputed analyses Model 1–Model 6.

Sensitivity analysis Model 1–Model 6.

## Footnotes

1 B = un-standardized beta.
